# Impact of sustained virologic response in regression of portal hypertension in Egyptian patients with hepatitis C virus-associated cirrhosis and portal hypertension

**DOI:** 10.1186/s43066-022-00188-x

**Published:** 2022-04-08

**Authors:** Heba Ahmed Faheem, Nannes Adel Abdulmeged, Hany Aly Hussein, Ahmed Abdelaziz Elmoursi, Heba T-allah Mohammed Yousry Elnaggar, Ramy Samir Ghait

**Affiliations:** 1 Internal Medicine and Hepatology, Gastroenterology, Faculty of Medicine, Ain Shams University, Cairo, Egypt; 2 Radiodiagnosis, Faculty of Medicine, Ain Shams University, Cairo, Egypt

**Keywords:** HCV-related liver cirrhosis, Clinically significant portal hypertension, Sustained virological response, Direct-acting antiviral therapy, Noninvasive fibrosis marker

## Abstract

**Background:**

Portal hypertension (PH) is a common consequence in hepatitis C virus cirrhotic patients. With interferon alpha-based therapy, SVR was linked to improved PH and fibrosis regression. SVR to oral antiviral regimens is linked to reduced portal pressure in patients with clinically significant portal hypertension (CSPH) at baseline. However, CSPH continues in most of the patients. This study aims to assess the reversibility and/or improvement of PH in Egyptian patients with HCV-related cirrhosis and CSPH after achieving SVR with DAAs. The second aim is to evaluate performance of the noninvasive markers of fibrosis in prediction of the presence and/or reversibility of the CSPH in correlation to radiological and endoscopic parameters.

**Subjects and methods:**

We evaluated noninvasive parameters, radiological and endoscopic signs of PH at baseline, and/or SVR 24 and SVR 48 post-DAA therapy in 40 patients with cirrhosis and CSPH (group A) and another 40 patients with cirrhosis only (group B).

**Results:**

In group A, the spleen diameter decreased from baseline (15.74 ± 1.53 cm), and SVR 24 (15.48 ± 1.51), to SVR 48 (15.35 ± 1.49 cm). No ascites detected at SVR 48 in 62.5%. Portal vein diameter and portal vein blood velocity reduced to 13.53 ± 1.07 mm and 14.14 ± 2.2 cm/s at SVR 48, with reversibility of hepatic vein waveform towards the triphasic pattern. Medium to large esophageal varices regressed from 52.5% to 2.5%, and up to 70% of patients showed no EVs at SVR 48. In group A, 24 patients showed complete reversibility of CSPH, and 16 patients showed improvement of CSPH. Child-Pugh score, FIB-4 index, King’s score, and Lok index revealed higher significance for detection of the presence of PH. Child-Pugh score, PC/SD ratio, and Lok index revealed higher significance for detection of reversibility of PH.

**Conclusion:**

We concluded that CSPH improved after SVR with DAAs and completely regressed in some patients. Upon predicting the presence of PH, Child-Pugh score, FIB-4 index, King’s score, and Lok index were the most significant noninvasive scores. While for predicting the reversibility of PH, Child-Pugh score, PC/SD ratio, and Lok index were the most significant scores.

## Background

Hepatitis C virus (HCV) is implicated in significant morbidity and mortality, mainly from portal hypertension (PH) complications [1]. The outcome of regression of liver fibrosis and clinically significant portal hypertension (CSPH) in patients with HCV-related compensated liver cirrhosis in response to newly developed direct-acting antivirals (DAAs) is still of interest by multiple studies as it has not yet been completely understood [2]. Recently most of studies showed significant improvement in liver function early after sustained virological response (SVR), persisted with long-term SVR, which is explained by rapid decreases in hepatic venous pressure gradient (HVPG) and liver stiffness; however, evidence is still scarce about this outcome with short-term follow-up [3].

Measurement of the hepatic venous pressure gradient (HVPG) remains the “gold standard” to measure portal pressure, but its invasiveness favor the use of noninvasive methods to predict the presence of CSPH after they have shown good correlation with liver histology [4].

In the current study, we *aim primarily* to assess the reversibility and/or improvement PH in Egyptian patients with HCV-related cirrhosis and CSPH after achieving SVR with DAAs, according to protocol approved by the National Committee for Control of Viral Hepatitis (NCCVH) in Egypt. The *second aim* is to evaluate performance of the noninvasive markers of fibrosis in prediction of the presence and/or reversibility of the CSPH in correlation to radiological and endoscopic parameters.

## Methods

This is a single-center case-control study which was conducted at Ain Shams University Hospitals, Hepatology Outpatient Clinic, Viral Hepatitis Treatment Unit, Endoscopy Unit and Radiodiagnosis Department, Faculty of Medicine, Cairo, during the period from March 2020 to June 2021.

### Patients

A sum of 80 patients with HCV-related chronic liver disease treated with DAAs were enrolled in the study.

*Diagnosis of liver cirrhosis* was made based on clinical features, e.g. (clubbing, palmar erythema, spider naevi, gynecomastia, female pubic hair pattern, distended abdominal veins, splenomegaly, or ascites), laboratory values (high INR, high total bilirubin, and low serum albumin), and abdominal US signs (shrunken or enlarged nodular liver with increased echo-texture, a blunt edge, and distorted architecture, splenomegaly, or ascites).

*Detection of CSPH* was made based on several radiological parameters such as splenic size and the presence of ascites detected by US (PVD, PVV, reversal of blood flow, and the presence of collaterals detected by PV duplex), as well as endoscopic signs using EGD to detect the presence or absence of EVs or fundal varices, and their grade, as well as the presence or absence of portal gastropathy and duodenopathy.

### Exclusion criteria

Patients previously treated with IFN-based therapy, liver cirrhosis from etiology rather than HCV infection, the presence of HCC, refusal to participate in the study, and pregnancy or lactating females were excluded.

### Study design

Our study was divided into two parts and two aims. In the first part we categorized the patients into two groups, (Group A), 40 patients with HCV related liver cirrhosis and CSPH and (Group B) 40 patients with HCV related liver cirrhosis without CSPH to identify our primary aim. In the second part, we further divide group A into two subgroups: [Group I (24 patients) - Reversible CSPH] and [Group II (16 patients) - Improved CSPH] to identify our second aim.

All patients were subjected to the following:

1. Full history and clinical evaluation
2. Laboratory tests (ALT, AST, total and direct bilirubin, serum albumin, INR, CBC, serum creatinine, and hepatitis serology (HBsAg and HCV Ab), and quantitative PCR for HCV) and abdominal US with comment on hepatic and splenic size and texture, and degree of ascites, were applied at the baseline and at 24 and 48 weeks after end of treatment (SVR 24 and 48).
3. PV duplex with comment on PVD, PVV, HVWF and collaterals, and EGD (to detect the presence of EVs and its grading) at the baseline and SVR 48 only.
4. Also, different noninvasive liver fibrosis scores were calculated.
5. Child-Pugh score by Charles G. Child [5]
6. AST/ALT ratio (AAR) by Sheth’s formula [6]
7. AST to platelet ratio index (APRI) by Wai’s formula [7]: AST/upper limit normal of AST/(platelet count × 10^9^/L) × 100
8. FIB-4 score by Sterling’s formula [8]: age (years) × AST/[(platelet count × 10^9^/L) × √ALT]
9. King’s score by Cross’s formula [9]: (age × AST × INR)/(platelet count × 10^9^ /L)
10. Lok’s Index by Lok’s formula [10]: (−5.56–0.00089 × PLT + 1.26 × AST/ALT + 5.27 × INR)
11. PC/SD ratio: platelet count/spleen diameter by Giannini’s formula [11]

### Statistical analysis

The collected data was revised, coded, tabulated, and introduced to a PC using (SPSS 25). Data was presented, and suitable analysis was done according to the type of data obtained for each parameter. Mean, standard deviation (±SD) ranges for parametric numerical data, whereas median and interquartile ranges (IQR) for nonparametric numerical data, as well as frequency and percentage of nonnumerical data. Analytical statistics were done by using Student *t*-test to assess the statistical significance of the difference between two study group means, Mann-Whitney test (*U*-test) to assess the statistical significance of the difference of a nonparametric variable between two study groups and chi-square test to examine the relationship between two qualitative variables, whereas Fisher’s exact test was used to examine the relationship between two qualitative variables when the expected count is less than 5 in more than 20% of cells. The ROC curve (receiver operating characteristic) provides a useful way to evaluate the sensitivity and specificity for quantitative diagnostic measures that categorize cases into one of two groups. The level of significance of *p*-value was detected with *p* > 0.05 being of nonsignificant (NS) value and *p* < 0.05 of significant (S) value.

## Results

### Baseline characteristics of groups A and B

As illustrated in Table 1, the studied groups showed no significant difference regarding the demographic data. However, there was statistically significant differences regarding serum albumin, INR, and platelet count. And the noninvasive serum markers include Child-Pugh score, PC/SD, FIB-4, King’s score, and Lok index. Concerning radiological and endoscopic parameters, they all showed significant difference between both groups.

**Table 1:** Baseline characteristic data of the studied groups

Variables		Group A	Group B	Test of significance	
		***N*** (%) Mean ± SD Median (IQR)	***N*** (%) Mean ± SD Median (IQR)	Value	***p***-Value
**Baseline demographic data**					
**Age**		55.98 ± 11.46	57.45 ± 10.21	***t =*** −0.608	0.545
**Gender**	**Male**	16 (40%)	12 (30%)	***X***^**2**^***=*** 0.879	0.348
	**Females**	24 (60%)	28 (70%)		
**SVR**	**SVR 12**	40 (100%)	40 (100%)	-	-
	**SVR 24**	40 (100%)	40 (100%)	-	-
**Child-Pugh score**	**Child A**	6 (15%)	40 (100%)	-	-
	**Child B**	34 (85%)	-	-	-
**Baseline laboratory values**					
**AST (IU/L)**		78.8 ± 46.53	83.7 ± 46.64	z = −0.390	0.697
**ALT (IU/L)**		55.78 ± 38.17	53.18 ± 30.4	z = −0.034	0.973
**T. bilirubin (mg/dL)**		1.6 ± 0.88	1.25 ± 0.5	z = −1.628	0.103
**Serum albumin (g/dL)**		2.88 ± 0.47	3.38 ± 0.45	t = −4.866	**< 0.001**
**INR**		1.35 ± 0.19	1.26 ± 0.16	t = 2.341	**0.022**
**Platelet count × 10**^**3**^**/mm**		80.5 ± 19.05	109.3 ± 13.41	t = −7.818	**< 0.001**
**Baseline noninvasive markers**					
**Child-Pugh classification**		7.68 ± 1.27	5.7 ± 0.46	t = 9.246	**< 0.001**
**APRI score**		2.77 ± 1.8	2.21 ± 1.3	z = −1.549	0.121
**AAR**		1.83 ± 2.07	1.68 ± 0.53	z = −1.126	0.260
**PC/SD ratio**		525 ± 166	828 ± 154	t = −8.467	**< 0.001**
**FIB-4 index**		8.19 ± 5.3	5.95 ± 1.9	z = −2.540	**0.011**
**King’s score**		76.99 ± 47.32	54.45 ± 30.06	z = −2.252	**0.024**
**Lok index**		3.16 ± 2.89	2.23 ± 0.99	z = −2.107	**0.035**
**Baseline abdominal US**					
**Liver texture**	Coarse	0 (0%)	12 (30%)	*X*^2^*=* 14.118	**< 0.001**
	Cirrhotic	40 (100%)	28 (70%)		
**Spleen diameter (cm)**		15.74 ± 1.53	13.34 ± 0.96	***t =*** 8.369	**< 0.001**
**Degree of ascites**	No	5 (12.5%)	40 (100%)	Fisher’s exact test	**< 0.001**
	Mild	15 (37.5%)	0 (0%)		
	Moderate	9 (22.5%)	0 (0%)		
	Tense	11 (27.5%)	0 (0%)		
**Baseline portal and hepatic veins duplex**					
**PV diameter (mm)**		14.41 ± 1.04	11.53 ± 1.42	***t =*** 10.343	**< 0.001**
**PV velocity (cm/s)**		12.44 ± 1.98	18.39 ± 1.76	***t =*** −14.201	**< 0.001**
**Hepatic vein waveform**	Monophasic	26 (65%)	0 (0%)	Fisher’s exact test	**< 0.001**
	Biphasic	8 (20%)	0 (0%)		
	Triphasic	6 (15%)	40 (100%)		
**Collaterals**	Yes	6 (15%)	0 (0%)	Fisher’s exact test	**0.026**
	No	34 (85%)	40 (100%)		

### Comparison between studied groups regarding DAA’s regimen

According to NCCVH 2020 Egyptian guidelines, 87.5% of group A patients received triple combination therapy of sofosbuvir, daclatasvir, and ribavirin, whereas 80% of group B received dual combination therapy of sofosbuvir and daclatasvir, without statistically significant difference between both groups. All the 80 patients completed their DAA’s regimen, and 100% of them achieved SVR at EOT and SVR 24 persisted to SVR 48 without reported adverse events, as shown in Tables 1 and 2.

**Table 2:** Direct-acting antivirals (DAAs) regimens in the studied groups

Treatment protocol	Groups		Fisher’s exact test	
	Group A	Group B		
	***N*** (%)	***N*** (%)	***p***-value	Sig.
**SOF/DAC**	1 (2.5%)	32 (80%)	0.557	NS
**SOF/DAC/RBV**	35 (87.5%)	4 (10%)		
**SOF/DAC/RBV/SIM**	2 (5%)	1 (2.5%)		
**PAR/OMB/RBV**	2 (5%)	2 (5%)		
**PAR/OMB/RBV/SOF**	0 (0%)	1 (2.5%)		

### Comparison between different intervals during DAAs in group A (at baseline, SVR 24, and SVR 48) regarding labs, scores, and abdominal US

Based on Table 3, there was significant improvement of all laboratory values, noninvasive scores, and abdominal US signs after DAAs, yet, degree of improvement was better early after SVR 24 in the 1st 24 weeks. Ascites has disappeared in 20 patients of group A population at SVR 48 13 patient of them were early after SVR 24.

**Table 3:** Pairwise comparison between baseline, SVR 24, and SVR 48 at group A regarding labs, scores, and abdominal US

Variables		Group A			Pairwise comparisons		
		Baseline	SVR24	SVR48	Mean difference (***p***-value)		
		Mean ± SD Median (IQR)	Mean ± SD Median (IQR)	Mean ± SD Median (IQR)	Baseline vs. SVR 24	SVR 24 vs. SVR 48	Baseline vs. SVR 48
**Laboratory values**							
**AST (IU/L)**		78.8 ± 46.53	41.7 ± 14.72	33.23 ± 5.73	−37.1 **(< 0.001)**	−8.48 **(0.004)**	−45.58 **(< 0.001)**
**ALT (IU/L)**		55.78 ± 38.17	34.9 ± 11.97	26.6 ± 6.77	−20.88 **(< 0.001)**	−8.3 **(< 0.001)**	−29.18 **(< 0.001)**
**T. bilirubin (mg/dL)**		1.6 ± 0.88	1.4 ± 0.65	1.29 ± 0.57	−0.2 **(< 0.001)**	−0.11 **(< 0.001)**	−0.31 **(< 0.001)**
**Serum albumin (g/dL)**		2.88 ± 0.47	3.15 ± 0.44	3.42 ± 0.33	0.27 **(< 0.001)**	0.27 **(< 0.001)**	0.55 **(< 0.001)**
**INR**		1.35 ± 0.19	1.3 ± 0.19	1.25 ± 0.17	−0.06 **(0.007)**	−0.05 **(0.047)**	−0.1 **(< 0.001)**
**Platelet count × 10**^**3**^**/mm**		80.5 ± 19.05	95.88 ± 20.99	100.95 ± 21.7	15.38 **(< 0.001)**	5.08 **(< 0.001)**	20.45 **(< 0.001)**
**Noninvasive scores**							
**Child-Pugh score**		7.68 ± 1.27	6.8 ± 1.14	6.5 ± 1.18	−0.88 **(< 0.001)**	−0.3 **(0.008)**	−1.18 **(< 0.001)**
**APRI score**		2.77 ± 1.8	1.24 ± 0.57	0.95 ± 0.37	−1.53 **(< 0.001)**	−0.29 **(< 0.001)**	−1.82 **(< 0.001)**
**AAR**		1.83 ± 2.07	1.25 ± 0.37	1.3 ± 0.26	−0.58 **(0.021)**	0.05 (1.00)	−0.54 **(0.07)**
**PC/SD ratio**		525 ± 166	636 ± 198	675 ± 200	112 **(< 0.001)**	38 **(< 0.001)**	150 **(< 0.001)**
**FIB-4 index**		8.19 ± 5.3	4.36 ± 1.68	3.79 ± 1.25	−3.84 **(< 0.001)**	−0.57 **(0.001)**	−4.4 **(< 0.001)**
**King’s score**		76.99 ± 47.32	33.03 ± 14.51	23.98 ± 8.08	−43.96 **(< 0.001)**	−9.05 **(< 0.001)**	−53.01 **(< 0.001)**
**Lok index**		3.16 ± 2.89	1.99 ± 1.23	1.77 ± 1.05	−1.17 **(< 0.001)**	−0.22 (0.169)	−1.39 **(< 0.001)**
**Abdominal US**							
**Spleen diameter (cm)**		15.74 ± 1.53	15.48 ± 1.51	15.35 ± 1.49	**< 0.001**	**< 0.001**	**0.003**
**Degree of ascites**	**No**	5 (12.5%)	18 (45%)	25 (62.5%)	**< 0.001**	**< 0.001**	**0.008**
	**Mild**	15 (37.5%)	18 (45%)	11 (27.5%)			
	**Moderate**	9 (22.5%)	4 (10%)	4 (10%)			
	**Tense**	11 (27.5%)	0 (0%)	0 (0%)			

### Comparison between different intervals during DAA’s in group A (at baseline and SVR 48) regarding portal, hepatic veins duplex, and EGD

There was significant improvement in (PV diameter), (PV velocity), and (HVWF) towards the triphasic pattern, as well as regression of EVs in (62.5 %) and disappearance of risky signs in (27.5 %) of group A population after DAA’s as shown in Table 4.

**Table 4:** Pairwise comparison between baseline and SVR 48 at group A regarding portal and hepatic vein duplex and EGD

Variables		Group A		Test of significance	
		Baseline	SVR 48		
		***N*** (%) Mean ± SD	***N*** (%) Mean ± SD	Test	***p***-value
**Portal and hepatic veins duplex**					
**PV diameter (mm)**		14.41 ± 1.04	13.53 ± 1.07	(Paired *t*-test) ***t =*** 7.991	**< 0.001**
**PV velocity (cm/s)**		12.44 ± 1.98	14.14 ± 2.2	(Paired *t*-test) ***t =*** −9.651	**< 0.001**
**Hepatic vein waveform**	**Monophasic**	26 (65%)	15 (37.5%)	Marginal homogeneity	**< 0.001**
	**Biphasic**	8 (20%)	14 (35%)		
	**Triphasic**	6 (15%)	11 (27.5%)		
**Collaterals**	**Yes**	6 (15%)	6 (15%)	McNemar test	1.00
	**No**	34 (85%)	34 (85%)		
**Endoscopy (EGD)**					
**EVs size**	**No**	3 (7.5%)	28 (70%)	Marginal homogeneity	**< 0.001**
	**Small**	16 (40%)	11 (27.5%)		
	**Medium**	15 (37.5%)	1 (2.5%)		
	**Large**	6 (15%)	0 (0%)		
**Risky signs of EVs**	**Yes**	11 (27.5%)	0 (0 %)	McNemar test	**0.001**
	**No**	29 (72.5%)	40 (100 %)		

### Comparison between two subgroups of group A (group I) and (group II) after DAA’s

Serum albumin, platelet count, Child-Pugh score, and PC/SD ratio were the only laboratory values and noninvasive serum markers beside the mentioned radiological signs on Table 5 that showed statistically significant difference between two subgroups of group A.

**Table 5:** Comparison between two subgroups of group A

Variables		(Group I)	(Group II)	Student ***t***-test	
		***N*** (%) Mean ± SD	***N*** (%) Mean ± SD	***T***-test	***p***-value
**Laboratory values**					
**AST (IU/L)**		88.5 ± 51.28	64.25 ± 34.95	−1.650	0.107
**ALT (IU/L)**		64.04 ± 38.46	43.38 ± 35.29	−1.719	0.094
**T. bilirubin (mg/dL)**		1.58 ± 0.97	1.63 ± 0.75	0.170	0.866
**Serum albumin (g/dL)**		3.08 ± 0.4	2.57 ± 0.4	−3.979	**< 0.001**
**INR**		1.32 ± 0.17	1.4 ± 0.22	1.320	0.195
**Platelet count × 10**^**3**^**/mm**		89.46 ± 17.89	67.06 ± 11.49	−4.822	**< 0.001**
**Noninvasive serum markers**					
**Child-Pugh score**		7.08 ± 1.25	8.56 ± 0.63	4.371	**< 0.001**
**APRI score**		2.86 ± 2.05	2.63 ± 1.37	−0.390	0.699
**AAR**		1.9 ± 2.65	1.74 ± 0.65	−0.230	0.819
**PC/SD ratio**		609 ± 156	398 ± 75	−5.694	**< 0.001**
**FIB-4 index**		8.07 ± 6.34	8.38 ± 3.39	0.179	0.859
**King’s score**		80.03 ± 53.02	72.42 ± 38.4	−0.493	0.625
**Lok index**		2.99 ± 3.61	3.42 ± 1.23	0.462	0.647
**Abdominal US**					
**Spleen diameter (cm)**		16.91 ± 0.82	14.96 ± 1.4	***t =*** 5.541	**< 0.001**
**Portal and hepatic veins duplex**					
**PV diameter (mm)**		13.94 ± 1.04	15.13 ± 0.53	***t =*** 4.755	**< 0.001**
**PV velocity (cm/s)**		13.44 ± 1.84	10.94 ± 1.01	***t =*** −5.522	**< 0.001**
**HV waveform**	**Monophasic**	1 (4.2%)	14 (87.5%)	Fisher’s exact test	**0.006**
	**Biphasic**	12 (50%)	2 (12.5%)		
	**Triphasic**	11 (45.8%)	0 (0%)		
**Collaterals**	**Yes**	1 (16.67%)	5 (83.33%)	Fisher’s exact test	**0.029**
	**No**	23 (67.65%)	11 (32.35%)		

### ROC curve of laboratory values, noninvasive markers, spleen diameter, and portal vein duplex in predicting the presence and reversibility of portal hypertension

**Fig. 1:**
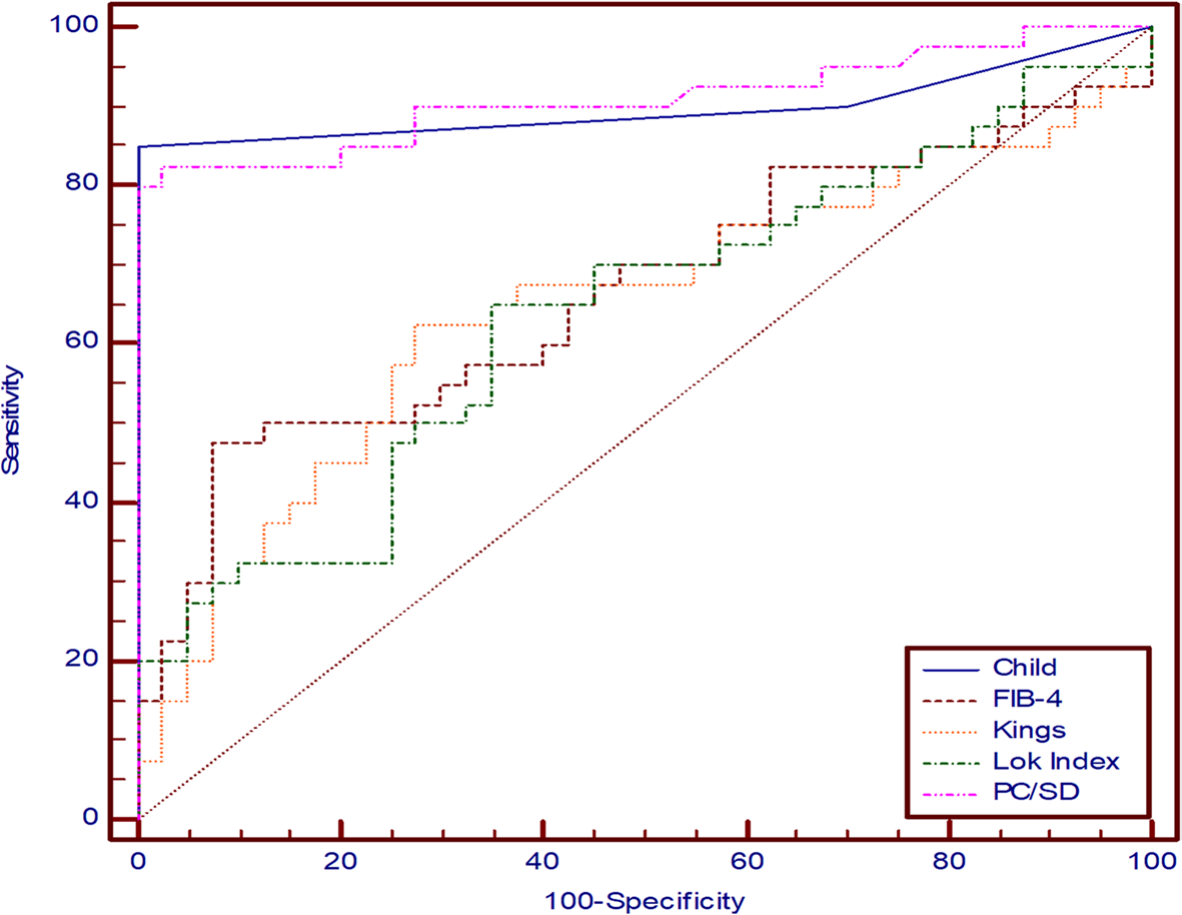
Prediction of the presence of CSPH in HCV-related liver cirrhosis

**Fig. 2:**
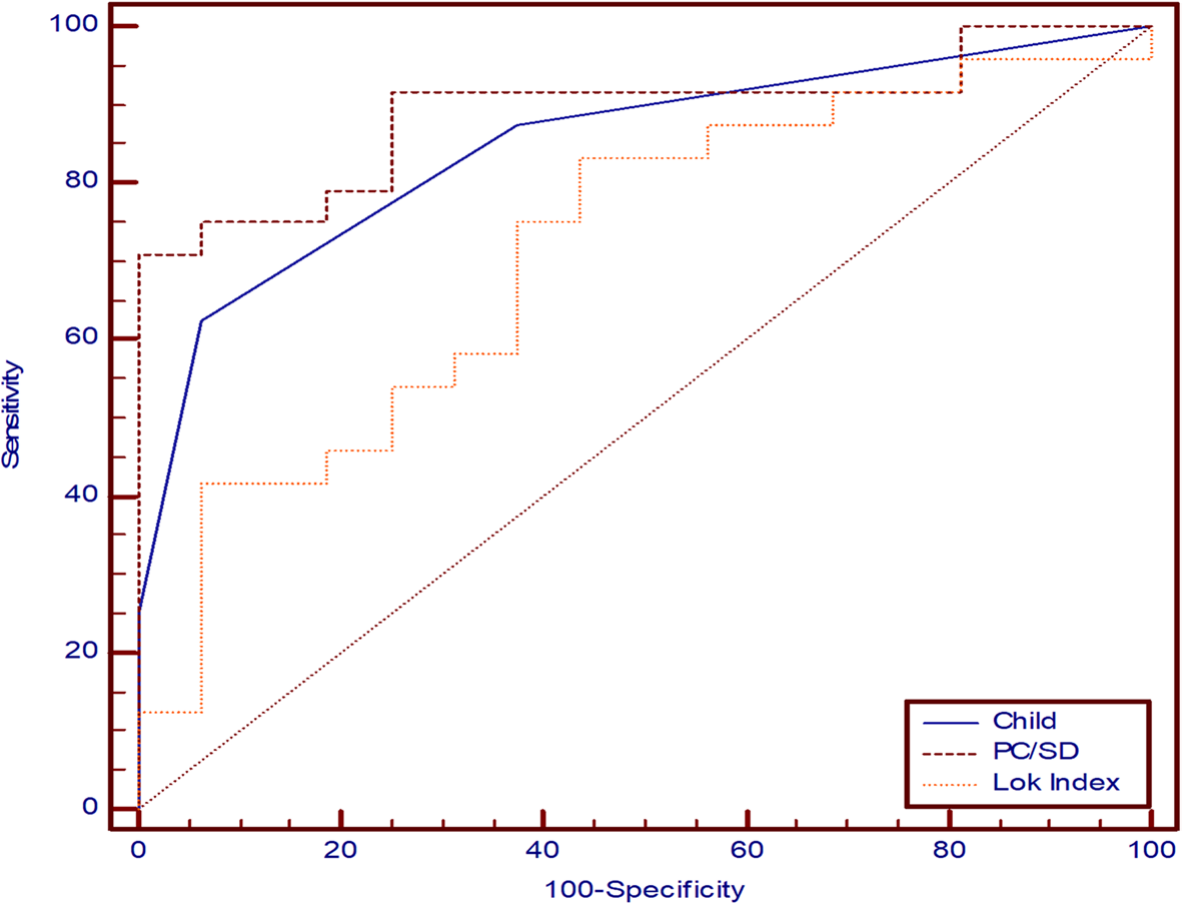
Prediction of portal hypertension reversibility in patients with HCV-related liver cirrhosis with CSPH

As regard prediction of the *presence* of PH, Child-Pugh score at cutoff value of > 6, PC/SD ratio at cutoff value of (≤ 6.69), FIB-4 index at cutoff value of > 8.01, King’s score at cutoff value of > 57.95, and Lok index at cutoff value of (> 2.61) were the most significant noninvasive scores, with (85%, 80%, 47.5%, 62.5%, and 65%) sensitivity, respectively, as shown on Table 6 and Fig. 1). While for predicting the *reversibility* of PH, Child-Pugh score at cutoff value of ≤ 7, PC/SD ratio at cutoff value of (> 5.24), and Lok index at cutoff value of (≤ 3.15) were the most significant scores, with 62.5%, 70.83%, and 83.33% sensitivity, respectively, as shown on Table 7 and Fig. 2.

**Table 6:** ROC curve for predicting the presence of CSPH in HCV-related liver cirrhosis

Variables	AUC	95% *CI*	Sig.	Cutoff value	Sensitivity	Specificity	PPV	NPV
**Laboratory values**								
**INR**	0.633	0.518 to 0.738	**0.033**	>1.17	87.50%	37.50%	58.3	75
**Platelet count**	0.887	0.797 to 0.947	**< 0.001**	≤ 98	82.50%	95%	94.3	84.4
**Noninvasive markers**								
**Child-Pugh score**	0.897	0.809 to 0.954	**< 0.001**	> 6	85%	100%	100	87
**PC/SD ratio**	0.909	0.824 to 0.962	**< 0.001**	≤ 669	80%	100%	100	83.3
**FIB-4 index**	0.665	0.551 to 0.767	**0.008**	> 8.01	47.50%	92.50%	86.4	63.8
**King’s score**	0.646	0.531 to 0.750	**0.022**	> 57.95	62.50%	72.50%	69.4	65.9
**Lok index**	0.637	0.522 to 0.742	**0.03**	> 2.61	65%	65%	65	65
**Portal vein duplex and spleen diameter**								
**PVD**	0.949	0.875 to 0.986	**< 0.001**	> 13.8	82.50%	95%	94.3	84.4
**PV velocity**	0.984	0.926 to 0.999	**< 0.001**	≤ 16.5	100%	85%	87	100
**Spleen diameter**	0.892	0.802 to 0.950	**< 0.001**	> 14.3	80%	87.50%	86.5	81.4

**Table 7:** ROC curve predicting reversibility of portal hypertension in patients with HCV-related cirrhosis with CSPH

Variables	AUC	95% ***CI***	Sig.	Cutoff value	Sensitivity	Specificity	PPV	NPV
**Laboratory values**								
**Platelet count**	0.853	0.705 to 0.945	**< 0.001**	> 73	83.33%	81.25%	87	76.5
**Noninvasive markers**								
**Child-Pugh score**	0.848	0.699 to 0.941	**< 0.001**	≤ 7	62.5%	93.75%	93.7	62.5
**PC/SD ratio**	0.891	0.751 to 0.967	**< 0.001**	> 524	70.83%	100%	100	69.6
**Lok index**	0.714	0.549 to 0.845	**0.012**	≤ 3.15	83.33%	56.25%	74.1	69.2
**Portal vein duplex and spleen diameter**								
**PVD**	0.863	0.718 to 0.951	**< 0.001**	≤ 14	50.00%	100%	100	57.1
**PVV**	0.9	0.763 to 0.972	**< 0.001**	> 11	91.67%	75%	84.6	85.7
**Spleen diameter**	0.868	0.724 to 0.954	**< 0.001**	≤ 15.5	66.67%	93.75%	94.1	65.2

## Discussion

Portal hypertension is the most common cause of complications in cirrhotic patients. While certain symptoms of it are obvious (e.g., ascites), others are more subtle. EVs, for example, are asymptomatic until they bleed. Patients with PH should be identified and offered endoscopic screening before bleeding develops. In addition to cirrhotic consequences, portal hypertension is linked to a higher risk of death in individuals with various liver diseases [4].

Our finding was roughly close to Abd El-Wahab et al. [12], who showed that males were 50.7% and females were 49.3%, whereas in our study, males represent 40% and 30% in group A and group B, and females represent 60% and 70% of groups A and B, respectively. Disparately, Mehrez et al. [13] studied 50 Egyptian patients with HCV genotype 4 infection and showed that 54% were males and 46% were females.

Regarding the mean age of patients, both were similar to our study; it was 52 years ± 10.3 at Abd El-Wahab et al. and 52.92 years at Mehrez et al., while for our patients, it was 55.98 ± 11.46 and 57.45 ±10.21 years in groups A and B, respectively.

All of our patients achieved successful HCV eradication with 100% SVR at EOT, persisted to SVR 24 and SVR 48. Giannini et al. [2], Ebeid et al. [14], and Przekop et al. [15], showed similar results by achieved SVR nearly in 100% of patients.

This study showed significant improvement of AST, ALT, serum albumin, INR, and platelet count, particularly between baseline and SVR 24, in all patients in the main groups of the study. This matched with Mehrez et al. [13] and Ippolito et al. [16], which followed their patients at SVR 12, and also Puente et al. [17], who followed the patients at week 8 during treatment and at SVR 72 posttreatment and reported significant improvement in all laboratory values with minor differences.

Another study by Elsharkawy et al. [18] showed significant improvement in AST, ALT, serum albumin, and INR, but it was insignificant for platelets and total bilirubin. The difference in comparison with other studies was pointed to efficacy of SOF/DAC combination in improving the liver necro-inflammatory more than SOF/SIM or SOF/RIB combination in cirrhotic patients, as only 23.5% of his study population received SOF/DAC, in contrast to our study where 92.5% has received SOF/DAC-based combinations.

This study is in concordance with Elsayed et al. [19], which was carried on 100 patients with chronic HCV-induced liver disease with early stage of cirrhosis, found a significant improvement at 6 months after DAAs (at SVR 24) in laboratory values (platelet count, albumin, bilirubin, AST, ALT), PVV, APRI score, and PC/SD ratio, with contrast to our results regarding PVD that was not significantly decreased after DAAs (*p*-value = 0.345). The difference between our findings and results of Elsayed et al. regarding PVD could be due to difference in inclusion criteria as we included patients with compensated liver cirrhosis and early CSPH, and majority of our patients were child B, while Elsayed et al. exclude them from his study. This explained the noticeable improvement in PVD and PVV in our results due to decrease in the intrahepatic pressure as a sequelae of improvement of necroinflammation by the DAAs.

Our results illustrated a significant difference between main groups of the study at baseline regarding Child-Pugh score, with mean ± SD for group A (7.68 ± 1.27) and for group B (5.7 ± 0.46). Also, Child-Pugh score showed significant difference in each group independently.

In group A, it improved from 7.68 ± 1.27 at baseline to 6.8 ± 1.14 at SVR 24 and to 6.5 ± 1.18 at SVR 48. However, this improvement was noticed to be more significant in the 1st 6 months early after DAA’s therapy. Furthermore, Child-Pugh score showed significant difference between group I and group II. These findings are in agreement with Ali et al. [20], who found significant improvement on Child-Pugh score from mean 7.3 to 5.9, and also Ji et al. [21], who found significant improvement on it from 6.30 ± 1.60 to 5.87 ± 1.14 at SVR 24. This findings was in concordance with Knop et al. [22] and Ippolito et al. [16]. This is referred to prevention of further stress on liver parenchyma by viral replication after eradication by DAAs.

Also, Knop et al. [22], Giannini et al. [2], and Cheng et al. [23] reported significant improvement of APRI score between baseline and SVR 24 in compensated HCV cirrhotic patients (*p* < 0.001*). These findings were in line with our results, in which there were significant improvement of all noninvasive scores after treatment with DAAs in both main study groups.

Regarding FIB-4 score, King’s score, and Lok index, they showed significant difference between both groups at baseline and significant improvement after DAA’s therapy. These results were similar to Abd El-Wahab et al. [12], with regard to King’s score; however, Lok’s index did not improve significantly after treatment (*p* = 0.987). Abd El-Wahab et al. suggested that these scores are affected by the variations in platelets count, AST, ALT, and ɣGT levels, and the resolution of established liver necroinflammation and fibrosis is a dynamic process may take several years. However, this remained controversial as other studies stated that the inflammatory activity did not contribute to liver stiffness.

About ascites detected by abdominal US, it has improved mainly between baseline and SVR 24. This study found that ascites has significantly resolved in group A 45% at SVR 24 versus 12.5% at baseline, with *p*-value ≤ 0.001*). This finding is close to Romano et al. [24], whereas ascites were resolved in 29% of patients 3 months posttreatment (65% versus 36%, *p*-value < 0.001*). Our finding suggest that DAAs can attenuate further hepatic decompensation by resolving ascites, and several studies have demonstrated that HCV patients who achieve SVR with DAAs experience significant improvements in their quality of life (social functioning, work productivity).

Regarding portal vein velocity, our results are in line with Soliman et al. [25], where PVV improved significantly from 11.889 ± 3.529 cm/s to 15.094 ± 4.250 cm/s, with (*p*-value ≤ 0.001*), as well as Mahmoud et al. [26], where PVV was increased significantly from 13.61 ± 2.53 cm/s at baseline to 14.72±2.67 cm/s at EOT and to 15.81±2.067 cm/s at SVR 48, with *p*-value ≤ 0.001*.

In our results, portal vein diameter showed 82.50% sensitivity and 95% specificity at cutoff value (> 13.8 mm), to predict the presence of CSPH in HCV-related liver cirrhosis, while showed 50% sensitivity and 100% specificity, at cutoff value (≤ 14 mm), to predict reversibility of CSPH. These findings are similar to Hagen-Ansert [27], who reported that a diameter (> 13 mm) was considered as a predictor of PH in patients with cirrhosis, but unlike Mahmoud et al. [26] and Mihai et al [28], as their results were insignificant regarding PVD.

Agha et al. [29] showed that PC/SD ratio had noninvasive relevance in diagnosis of EVs in a large population of HCV-infected cirrhotic patients; although previous studies limited their efficacy only to detect the presence of EVs, our study showed that it is still a useful noninvasive tool for the detection of CSPH in patients with HCV-related liver disease, as it is cheap, accurate, and easy applicable tool especially in developing countries where endoscopies are costly. This study showed that regarding prediction of CSPH in HCV-related liver disease, PC/SD ratio has the advantage, followed by Child score and platelet count as a most significant parameters; however, newly developed scores as FIB-4 index, King’s score, and Lok index were significant also with AUROC 0.665, 0.646, and 0.637, respectively.

Our findings concerning the best performing noninvasive serum biomarker of liver fibrosis in prediction of the presence of CSPH in HCV-related liver cirrhosis using the ROC curve (AUROC) were the FIB-4 score (*AUROC* = 0.665; *PPV* = 86.4%; *NPV* = 63.8%), followed by King’s score (*AUCROC* = 0.637; *PPV* = 69.4%; *NPV* = 65.9%), and then Lok index (*AUROC* = 0.665; *PPV* = 65%; *NPV* = 65%). This was in line with Ishida et al. [30], who found that FIB-4 score provided the greatest diagnostic accuracy in predicting both EVs and CSPH.

The current study was in agreement with Abd El-Wahab et al. [12], as regard best performing test for prediction of the presence of CSPH in HCV-related liver cirrhosis was for FIB-4 (*AUROC* = 0.791; *CI* = 73.4%–84.8%), followed by King’s score (*AUCROC* = 0.786; *CI* = 72.7%–84.5%), and then Lok index (*AUROC* = 0.762; *CI* = 69.9%–82.5%), but they were not in agreement regarding APRI score.

In accordance with Wang et al. [31], King’s score and Lok index were exhibited the best performance, as indicated by AUROCs of 0.755 and 0.740, respectively, although performed on different etiologies causes liver fibrosis, and also, combination between King’s and Lok index may be used as an initial screening tool to identify cirrhosis patients who are at very high risk of CSPH and to determine the need for further evaluation, but they were not in agreement regarding APRI score.

### Limitation

The limitations of our study were its relatively small sample size due to covid-19 precautionary measures which limited the endoscopy and ultrasonography lists and made many patients to miss their follow-up appointments. Also, being only a single-center experience was a week point.

On the other hand, our study had relatively longer period of follow-up with combination of various important tools including noninvasive serum biomarkers, ultrasonography and duplex studies, and endoscopic evaluation, so the results may differ from those in previous publications.

## Conclusion

Sustained virological response in the current study occurs in all patients with HCV-related cirrhosis who were treated with different DAAs regimens, regardless severity of cirrhosis, including those traditionally considered “difficult to treat.”

We also conclude that CSPH improves after cure of HCV infection by DAAs and completely regress in some patients, which is accompanied by improvements in noninvasive parameters of liver fibrosis and liver function, as well as a decrease in parameters suggestive of portal hypertension. In addition, we noticed accuracy of these noninvasive serum markers of fibrosis in prediction of the presence or reversibility of CSPH.

### Recommendation

Further studies are needed to confirm and clarify these initial observation and data between noninvasive serum markers, stiffness measurement modalities, and invasive ones, e.g., HVPG. As well as implication of these markers in the clinical practices as screening tool for PH, especially in the current situation of rising prevalence of liver cirrhosis cases due to MAFLD, without availability of cost-effective tool of screening for presence of and / or reversibility of liver injury as well as PH if present.

## Data Availability

The datasets used and/or analyzed during the current study are available from the corresponding author on a reasonable request.

## Abbreviations

ALT: Alanine aminotransferase
AST: Aspartate aminotransferase
AAR: AST/ALT ratio
AFP: Alfa-fetoprotein
APRI: AST to platelet ratio index
AUC: Area under the curve
CI: Confidence interval
CSPH: Clinically significant portal hypertension
DAAs: Direct-acting antivirals
DAC: Daclatasvir
EGD: Esophagogastroduodenoscopy
EOT: End of treatment
EVs: Esophageal varices
FIB-4: Fibrosis index based on four factors
HBsAg: Hepatitis B surface antigen
HCC: Hepatocellular carcinoma
HCV: Hepatitis C virus
HCV Ab: Hepatitis C virus antibody
HVPG: Hepatic venous pressure gradient
HVWF: Hepatic venous waveform
INR: International normalization ratio
IQR: Interquartile range
NCCVH: National Committee for Control of Viral Hepatitis
NPV: Negative predictive value
NS: Nonsignificant
OMB: Ombitasvir
PAR: Paritaprevir
PC: Personal computer
PCR for HCV: Polymerase chain reaction for hepatitis C virus
PC/SD: Platelet count/spleen diameter
PH: Portal hypertension
PPV: Positive predictive value
PT: Prothrombin time
PTT: Partial thromboplastin time
PV: Portal vein
PVD: Portal vein diameter
PVV: Portal vein velocity
RBV: Ribavirin
ROC: Receiver operating characteristic
S: Significant
SD: Standard deviation
Sig: Significant
SIM: Simeprevir
SOF: Sofosbuvir
SPSS: Statistical Package for Social Science
SVR: Sustained virological response
US: Ultrasonography
